# Microsurgical obliteration of a superior petrosal dural arteriovenous fistula presenting with a symptomatic brainstem cavernous malformation

**DOI:** 10.3171/2025.7.FOCVID2553

**Published:** 2025-10-01

**Authors:** Ryan M. Naylor, Alexander Pais, Stephen Graepel, Giuseppe Lanzino, Michael J. Link

**Affiliations:** Department of Neurosurgery, Mayo Clinic, Rochester, Minnesota

**Keywords:** cerebrovascular neurosurgery, skull base neurosurgery, dural arteriovenous fistula, cavernous malformation, retrosigmoid craniotomy

## Abstract

In this video, the authors present the microsurgical treatment of a superior petrosal dural arteriovenous fistula (dAVF) associated with a symptomatic hemorrhagic brainstem cavernous malformation in a 38-year-old male. The patient underwent a retrosigmoid craniotomy for cavernoma evacuation and fistula disconnection. Intraoperative indocyanine green angiography revealed cortical venous reflux not evident on catheter angiography, prompting reclassification to a higher-grade fistula. Postoperatively, the patient returned to neurological baseline, and follow-up imaging confirmed complete fistula obliteration, cavernoma resection, and new appearance of a developmental venous anomaly (DVA). This case highlights the dynamic nature of dAVF venous drainage and illustrates the rare co-occurrence of dAVF, DVA, and brainstem cavernoma.

The video can be found here: https://stream.cadmore.media/r10.3171/2025.7.FOCVID2553

## Transcript

Here we present the microsurgical obliteration of a superior petrosal dural arteriovenous fistula presenting with a symptomatic brainstem cavernous malformation.

### 0:29 History of Present Illness and Physical Examination.

The patient was a healthy 38-year-old male who experienced abrupt onset of left-sided facial numbness, diminished hearing, imbalance, and dull headache. His neurological examination was normal except for left V2 distribution numbness.

### 0:43 Audiogram.

The patient’s audiogram demonstrated class A hearing in both ears.

### 0:48 Neuroimaging Findings.

MRI demonstrated a hemorrhagic lesion in the left ventrolateral pons and middle cerebellar peduncle that came to the surface of the brainstem. Notably, the hematoma was entirely intraparenchymal without any subdural or subarachnoid components. The radiographic differential diagnosis included hematoma, tumor, and cavernous malformation.

Catheter angiography demonstrated a dural arteriovenous fistula adjacent to the hemorrhagic brainstem lesion. The fistula drained into the left superior petrosal sinus and was supplied by the left meningohypophyseal trunk and petrosal branch of the left middle meningeal artery.^1^ The left sigmoid sinus was not patent, and XperCT shows the site of the fistula’s connection in relation to the petrous apex of the temporal bone.

### 1:33 Rationale for Procedure.

Although this was a low-grade Borden type I,^2^ Cognard type I^3^ fistula based on the angiogram, we felt that treatment was indicated based on the patient’s new neurological symptoms that corresponded to the hemorrhagic brainstem lesion as well as the adjacent fistula that was in very close proximity. Treatment was further supported based on the patient’s young age and excellent overall health.

Notably, patients with dural AV fistulas who present with hemorrhage or new neurological symptoms have an annual rate of hemorrhage and new or progressive neurological deficit between 7% and 19% as well as an annual mortality rate of 4%, highlighting the importance of timely intervention for these patients.^4^

We felt that microsurgery using a retrosigmoid craniotomy was the most appropriate treatment option in this case given the familiar and straightforward approach, the ability to decompress and possibly resect the hemorrhagic brainstem lesion, and the high likelihood of obliterating the fistula.^1,5,6^ We did not consider radiosurgery to be a great option in this case given the indeterminate nature of the symptomatic brainstem lesion and the associated latency period prior to fistula obliteration. Additionally, endovascular embolization would not address the brainstem lesion and was felt to be unacceptably risky in terms of treating the fistula given the arterial supply from the petrosal branch of the middle meningeal artery, which contributes to the vasovasorum of the facial nerve.^7^ Distal embolization of the petrosal branch could infarct the facial nerve and cause complete facial paralysis.

### 2:58 Description of Setup.

Our surgical setup included placing the patient in the right lateral decubitus position to expose the left suboccipital region and immobilizing the patient’s head in a Mayfield holder.

### 3:08 Necessary Equipment.

In addition to standard microsurgical instruments, necessary equipment includes an operative microscope equipped with the ability to visualize indocyanine green, temporary aneurysm clips, and Weck Horizon clips. We chose to perform fairly extensive neuromonitoring given the ambiguity of the brainstem lesion, and we monitored cranial nerves IV, V, VI, VII; brainstem auditory evoked responses; the laryngeal adductor reflex; and cranial nerve XI.

### 3:36 Key Surgical Steps.

Key surgical steps include a standard left-sided retrosigmoid craniotomy^8^ that is flush with the inferior edge of the transverse sinus and medial edge of the sigmoid sinus.

We then open the inferior portion of the dura first and release CSF to facilitate cerebellar relaxation and then complete a trap door–style dural opening that is pedicled onto the sigmoid sinus. The dura is protected from the light of the microscope with a moist Telfa and tacked up using 3-0 silk sutures to maximize the view of the CP angle.

Next, we perform extensive arachnoid dissection to expose the neurovascular contents of the CP angle, which allows the cerebellum to gently fall away without the use of fixed retractors. ICG angiography is performed, the fistula is obliterated, and the contents of the brainstem lesion are evacuated.

### 4:27 Operative Video.

We took the patient to the operating room and performed a left retrosigmoid craniotomy.

The arachnoid of the cerebellopontine angle is released beginning over the lower cranial nerves.

Just above the seventh and eighth cranial nerve complex, the subarcuate artery is identified, ligated, and divided. An arterialized vein emanating from the petrous dura was also coagulated and divided.

Arachnoid dissection continues over the eighth cranial nerve and between cranial nerve V and the VII/VIII complex, revealing the hemorrhagic brainstem lesion seen on MRI.

Here the fifth nerve is seen draped over the lesion.

The superior petrosal vein is seen as an arterialized stump with two narrow arterialized conduits emanating from it, which was suggestive of cortical venous reflux and venous outflow obstruction.

Indeed, ICG angiography demonstrated illumination of the superior petrosal vein during the arterial phase with cortical venous reflux.^9^ This drainage pattern was not appreciated on the preoperative angiogram, and based on the intraoperative ICG angiography, the fistula is better classified as a Borden type II, Cognard IIa+b fistula.^2,3^

The fourth nerve and motor root of the fifth nerve are identified.

Next, the arterialized draining veins are occluded using temporary clips to ensure that the fistula doesn’t swell or hemorrhage before permanent disconnection. The draining veins are then obliterated using bipolar electrocautery and divided.

The brainstem lesion is then entered. A pair of microscissors is used to open the capsule, and the hemorrhagic contents are evacuated using a combination of suction and microforceps. We took great care to avoid excessive manipulation of the hemosiderin-stained brainstem to avoid its injury. Our impression was that the lesion was a hemorrhagic brainstem cavernoma, and this was supported with pathological analysis.

We then proceeded with ligation of the superior petrosal sinus in order to achieve complete obliteration of the fistula and definitive cure for the patient. Here, the vein and tentorium are heavily coagulated.

The tentorium was incised with an 11 blade knife, and microscissors were used to circumferentially disconnect the superior petrosal sinus at the junction with the superior petrosal vein.^9^ It is important to map the course of the fourth nerve and note its entrance into the tentorium prior to disconnecting the superior petrosal sinus within the tentorium.

Brisk arterial bleeding is seen when the superior petrosal sinus is encountered, and this bleeding was controlled using bipolar electrocautery.

The entire stump of the superior petrosal vein is excised from the tentorium to ensure the complete disconnection of the superior petrosal sinus.

Finally, a Weck clip is placed across the location of the superior petrosal sinus in the tentorium, again to ensure its disconnection and hemostasis.

### 7:41 Brief Review of Clinical and Imaging Outcomes.

The patient awoke from surgery at his neurological baseline and had an uncomplicated postoperative course. He discharged home on postoperative day 3. At the 3-month follow-up visit, the patient reported that his preoperative facial numbness had completely resolved. He was otherwise asymptomatic and neurologically intact.

Postoperative head CT demonstrated appropriate evacuation of the contents of the brainstem cavernoma and Weck clip placement across the superior petrosal sinus at the petrous apex. Postoperative angiography showed complete obliteration of the fistula.

MRI performed at the 3-month follow-up showed complete removal of the hemorrhagic brainstem cavernoma without recurrent or residual disease. This MRI also demonstrated a large developmental venous anomaly running through the brainstem adjacent to the site of the prior cavernous malformation, which was not visualized on the initial MRI.

### 8:32 Learning Points.

A few take-home points from this unique case. First, developmental venous anomalies have been reported in association with cavernous malformations and dural AV fistulas. While this case highlights the rare co-occurrence of all three of these vascular anomalies, the presence of a shared pathological mechanism remains uncertain.^10^ Second, it is interesting that the preoperative angiogram failed to demonstrate cortical venous drainage through the superior petrosal vein, yet this was clearly seen intraoperatively using ICG angiography. It is important to appreciate the dynamic nature of dural AV fistulas, which may change their venous drainage based on regional changes in venous outflow patterns.^4^ And third, when disconnecting the superior petrosal sinus within the tentorium,^9^ it is important to visualize the entry point of the fourth nerve going into the tentorium to avoid injuring the nerve.

## Acknowledgments

ChatGPT was used to improve the clarity, readability, and grammar of the abstract.

## Disclosures

Dr. Lanzino reported consulting for Superior Medical Editors and Nested Knowledge.

## Author Contributions

Primary surgeon: Link. Assistant surgeon: Naylor, Pais, Lanzino. Editing and drafting the video and abstract: Link, Naylor, Pais. Critically revising the work: Link, Naylor, Lanzino. Reviewed submitted version of the work: Link, Naylor, Pais, Lanzino. Approved the final version of the work on behalf of all authors: Link. Supervision: Lanzino. Medical illustrator: Graepel.

## Supplemental Information

### Previous Presentations

North American Skull Base Society Meeting, New Orleans, LA, February 13–16, 2025.

### Patient Informed Consent

The necessary patient informed consent was obtained in this study.
